# β3-adrenergic receptor on tumor-infiltrating lymphocytes sustains IFN-γ-dependent PD-L1 expression and impairs anti-tumor immunity in neuroblastoma

**DOI:** 10.1038/s41417-023-00599-x

**Published:** 2023-02-28

**Authors:** Gennaro Bruno, Nicoletta Nastasi, Angela Subbiani, Alessia Boaretto, Sara Ciullini Mannurita, Gianluca Mattei, Patrizia Nardini, Chiara Della Bella, Alberto Magi, Alessandro Pini, Emanuela De Marco, Annalisa Tondo, Claudio Favre, Maura Calvani

**Affiliations:** 1 Department of Pediatric Hematology-Oncology, A. Meyer Children’s Hospital IRCCS, Florence, Italy; 2 Department of Health Sciences, University of Florence, Florence, Italy; 3 Department of Information Engineering, University of Florence, Florence, Italy; 4 Department of Experimental and Clinical Medicine, University of Florence, Florence, Italy; 5 Pediatric Hematology and Oncology, University Hospital of Pisa, Pisa, Italy

**Keywords:** Cancer microenvironment, Paediatric cancer, Tumour immunology

## Abstract

Neuroblastoma (NB) is a heterogeneous extracranial tumor occurring in childhood. A distinctive feature of NB tumors is their neuroendocrine ability to secrete catecholamines, which in turn, *via* β-adrenergic receptors ligation, may affect different signaling pathways in tumor microenvironment (TME). It was previously demonstrated that specific antagonism of β3-adrenergic receptor (β3-AR) on NB tumor cells affected tumor growth and progression. Here, in a murine syngeneic model of NB, we aimed to investigate whether the β3-AR modulation influenced the host immune system response against tumor. Results demonstrated that β3-AR antagonism lead to an immune response reactivation, partially dependent on the PD-1/PD-L1 signaling axis involvement. Indeed, β3-AR blockade on tumor-infiltrating lymphocytes (TILs) dampened their ability to secrete IFN-γ, which in turn reduced the PD-L1 expression, caused by TILs infiltration, on NB tumor cells. Further investigations, through a genomic analysis on NB patients, showed that high ADRB3 gene expression correlates with worse clinical outcome compared to the low expression group, and that ADRB3 gene expression affects different immune-related pathways. Overall, results indicate that β3-AR in NB TME is able to modulate the interaction between tumor and host immune system, and that its antagonism hits multiple pro-tumoral signaling pathways.

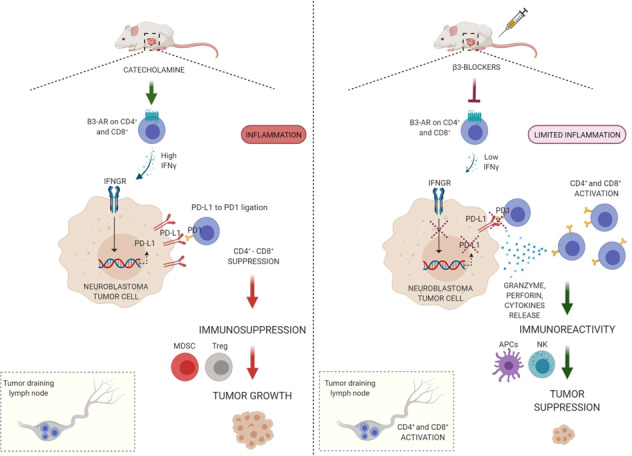

## Introduction

Neuroblastoma (NB) is a complex and heterogeneous disease, and the most common type of cancer diagnosed in the first year of life. NB is a neuroendocrine tumor that arises from committed precursor cell of the neural crest during the development of the sympathetic nervous system (SNS). This distinctive origin determines tumor localization, which usually occurs in the adrenal gland and/or sympathetic ganglia [1]. Clinical behavior of NB is highly variable, ranging from tumors that spontaneously regress, to others that became refractory to all available therapies including intensive chemotherapy, surgery, radiotherapy, autologous stem cell transplantation, and administration of retinoids (13-cis-retinoic acid) [2]. Alternative therapeutic targets for these unresponsive tumors are therefore needed.

Immunotherapy based on immune checkpoint inhibitors (ICIs) represents the most promising strategy for several solid tumors [3]. Among others, the programmed death ligand-1/programmed death-1 (PD-L1/PD-1) signaling axis impairs effector T cell immunity and enhances immune tolerance of tumor cells, playing a pivotal role in attenuating anti-tumor responses in both mouse and human cancers [4, 5]. However, while numerous studies have already investigated mechanisms underlying PD-1/PD-L1 signaling in adult cancer, little is known in pediatric tumors.

A complex cross-talk between the SNS and the immune system, via the catecholamine release and activation of the β-adrenergic receptors signaling, is able to determine the fate of tumor progression in many tumors [6]. This could be particularly true in NB patients in which, indeed, elevated level of catecholamines reflect the severity of malignancy [7].

The β3-adrenergic receptor (β3-AR) subtype has been recently identified as a critical regulator of cancer [8–10]. In particular, its expression and activation, both on tumor and stromal cells of the tumor microenvironment (TME), elicited pro-tumoral signaling pathways responsible of tumor progression in different pre-clinical models [11–14].

Here, in a syngeneic murine model of NB, we demonstrated that β3-AR on tumor-infiltrating lymphocytes (TILs) sustains an IFN-γ-dependent PD-L1 upregulation on tumor cells, which in turn lead to an immune-suppressive TME responsible for tumor escape. In addition, β3-AR antagonism was able to increase the number of immune-reactive CD8^+^, natural killer (NK) and dendritic cells (DCs), and to decrease the number of immune-suppressive regulatory T cells (Treg) and Myeloid-derived suppressor cells (MDSC) in TME. Furthermore, a Kaplan–Meier analysis showed that high *ADRB3* gene expression was associated with poor survival of NB patients, and analyses of different available public datasets of NB expression revealed a significant correlation between the *ADRB3* gene expression level and genes involved in signaling pathways related to the immune system/tumor interaction.

## Methods

### Cell culture

Neuro-2A murine NB cancer cells were obtained from ATCC (CCL-131) and were grown in DMEM supplemented with 10% of FBS, 2 mM L-glutamine, 100 U·ml^−1^ penicillin and 100 μg·ml^−1^ streptomycin at 37 °C in water-saturated, 5% CO_2_ atmosphere. Neuro-2a cells were routinely tested for mycoplasma contamination.

### Tumor syngeneic murine model

Female NCI A/JCr mice 4-week old were bought from Charles River Laboratories (Frederick). Neuro-2a cells (N2A) were subcutaneously implanted in A/J recipient mice by injecting 1 × 10^6^ cells in 100 μl of PBS in the right flank. When N2A cells formed a palpable tumor (about 8 days), treatments started. Treatments were administered twice a day for SR59230A (Tocris Bioscience) and vehicle (physiological solution), and at day 8 and day 12 for αPD-L1 antibody (InVivoMAb anti-mouse PD-L1 (B7-xhH1) #BE0101, Bio X Cell) and isotype control (InVivoMAb rat IgG2b isotype control # BE0090, Bio X Cell). IgG2b isotype control administration produced the same results of vehicle hence only vehicle condition is shown in figures. SR59230A was delivered at 10 mg/kg in physiological solution via intraperitoneal (i.p.); 250 µg of αPD-L1 was delivered i.p. in 100 µl of InVivoPure™ pH 6.5 Dilution Buffer (#IP0065); 250 µg of IgG2b isotype control mouse was delivered i.p. in 100 µl of InVivoPure™ pH 7.0 Dilution Buffer (#IP0070). Treatment with FTY720 (#6176, Tocris Biotechne) was administered at 2 mg/kg per os (p.o.) from day 6. Tumor growth rate was evaluated by measuring tumor mass with a caliber, and tumor mass volume calculated as volume = [(length × width)^2^/2]. Mice were sacrificed after 8 days of treatment.

### Western blot analysis

Cells were collected and lysed in RIPA buffer containing protease inhibitor cocktail. After quantification, 20 μg of total proteins were used to perform an SDS-PAGE and WB analysis. PVDF membranes were incubated overnight with the primary antibodies anti-PD-L1 (ab238697, Abcam), phospho-CREB (ab32096, Abcam), CREB (ab32515, Abcam), p-PKAα/β/γ (sc-32968, Santa Cruz Biotechnology), β-actin (sc-1615, Santa Cruz Biotechnology), β3-AR (ab94506, Abcam) at 4 °C and then with specific secondary antibodies for 1 h at room temperature. Binding of the antibodies with the specific proteins has been detected by using Clarity Western ECL Substrate (Bio-Rad) and images were acquired through the Chemidoc Imaging System (Biorad®). IMAGEJ software was used to perform quantitative analyses, and the signal intensity of the bands was expressed as fold-increase relative to control values.

### Co-culture for PD-L1 cytofluorimetric assay

Tumor cells were seeded on MW24 (45,000 cells/well) and after 24 h were co-cultured with lymphocytes, obtained from TDLNs of mice pre-treated for 2 days with Vehicle or SR59230A (T2), in a seeding ratio of 1:10 (tumor/lymphocytes). At the end of the total incubation period (48 h), lymphocytes were withdrawn together with the medium of the co-culture by gentle pipetting, and then tumor cells detached by trypsinization and marked with the anti-PD-L1 antibody (CD274 Antibody 12-5982-82, eBioscience™) for cytofluorimetric quantification. Stained cells were acquired on a MACSQuant Analyzer 10 Flow Cytometer (Miltenyi Biotec, Gladbach, Germany), and data were processed using Flowlogic software (Miltenyi Biotec, Gladbach, Germany).

### Co-culture for PD-L1 immunofluorescence and IFN-γ quantification

Tumor cells were seeded on microscope slides (20,000 cells/well) in Nunc Lab-Tek chamber slide 4, and after 24 h were co-cultured with lymphocytes, obtained from TDLNs of mice treated for 2 days with Vehicle or SR59230A (T2), in a seeding ratio of 1:10 (tumor/lymphocytes). After 48 h, lymphocytes in culture medium were collected by gentle pipetting, and then discarded through centrifugation; supernatant was used to quantify IFN-γ through Luminex xMAP technology according to the manufacturer’s instructions. Briefly, IFN-γ quantification in co-culture medium was performed using Bio-Plex Pro Mouse Cytokine IFN-γ Set (#171G5017M, Bio-Rad) and data were acquired on MAGPIX System (Luminex, Texas, USA). Analysis was performed using the Bio-Plex Manager™ software (BIO-RAD, California, USA).

Tumor cells on microscope slides were instead used to perform the PD-L1 immunofluorescence assay as follows. For immunofluorescence analysis cells were fixed in 3% paraformaldehyde in PBS for 20 min. Permeabilization was obtained by adding a solution containing Tween-20 0.5% in PBS for 30 min at room temperature. Cells were then blocked in 3% BSA for 1 h and incubated with primary antibody anti-PD-L1 (ab238697, Abcam) for 2 h. Subsequently, after washes in PBS, slides were incubated with Alexa-fluor 488 secondary antibody for 1 h. Images were obtained using a fluorescence microscope (DMi8 - Leica microsystems).

### siRNA electroporation of lymphocytes for β1- β2- and β3-AR silencing

Lymphocytes obtained from TDLNs were transfected with siRNAs for selective β1- (SASI_Mm01_00090154, sequence: GUGAUCGUGGCCAUCGCCA, Merck), β2- (SASI_Mm01_00154297, sequence: CCAAGUUCGAGCGACUACA, Merck) and β3-AR (SASI_Mm01_00145466, sequence: CAGUGGACGUGCUCUGUGU, Merck) silencing. Briefly, lymphocytes were electroporated with siRNAs (100 nM) using the 4D-Nucleofector X Unit (Lonza) and the P3 Primary Cell 4D-NucleofectorTM X Kit (V4XP-3024, Lonza). The DN-100 program, specific for murine T cell electroporation, was performed. After electroporation, lymphocytes were cultured in TexMACS Medium (130-097-196, Miltenyi Biotec) for 24 h and then co-cultured with N2A tumor cells for 48 h, as previously described, for PD-L1 quantification. β-ARs silencing efficiency in lymphocytes was evaluated through digital droplet PCR as follows.

### Digital droplet PCR for β-ARs mRNA quantification

RNA was reverse transcribed using iScript Advanced cDNA Synthesis Kit (Bio-Rad). ddPCR was performed using ddPCR Supermix for Probes (No dUTP) (Bio-Rad), according to manufacture instructions, on a QX200 system (Bio-Rad) and a C1000 Touch Thermal Cycler (Bio-Rad). The assays used were: Adrb3-FAM (dMmuCPE5088688), Adrb2-FAM (dMmuCPE5089938), Adrb1-FAM (dMmuCPE5100184), Rps18-HEX (dMmuCPE5196447). ddPCR data were analyzed using QuantaSoft™ software (version 1.7.4, Bio-Rad).

### RNA-sequencing

Excised tumors were placed in buffer RLT (Qiagen), and mechanically homogenized with gentleMACS Octo Dissociator (Miltenyi Biotec, Gladbach, Germany) using gentleMACS M Tubes (Miltenyi Biotec, Gladbach, Germany). Total RNA extraction was performed with the RNeasy Plus Mini kit (Qiagen), following the manufacturer’s instruction. RNA quantification was carried out using both NanoDrop 2000/2000c Spectrophotometers (ThermoFisher) and Qubit RNA High Sensitivity (HS) (ThermoFisher). RNA integrity was assessed using Agilent RNA 6000 Pico Kit on an Agilent Bioanalyzer and all samples with a RNA integrity number (RIN) lower than 6.00 were excluded.

Library preparation was performed using the TruSeq Stranded Total RNA Library Prep Gold Kit (Illumina) according to manufacturer’s instruction, with initial input of 500 ng of total RNA. The library quality was assessed using Agilent DNA 7500 kit on an Agilent Bioanalyzer, followed by library quantification using 1X dsDNA High Sensitivity (HS) (ThermoFisher). Libraries were sequenced on a NextSeq 550 Sequencing System (Illumina) using NextSeq 500/550 v2.5 (150 cycles) High Output Reagent Kits (Illumina). Runs were performed with a 2 × 76 bp configuration.

### mRNA quantification and transcriptomics analyses

Salmon (v. 1.5.1) in mapping-based mode was used to perform the quantification of the transcripts, and the mus musculus’ reference transcriptome GRCm38.98. The resulting *qf* files were analyzed by the statistical language R. The *tximport* package was used to import the data in R, and the transcripts were collapsed on to genes by *AnnotationDbi* package and *org.Mm.eg.db* database. Finally we performed the statistical analyses with *DESeq2*. We performed an Over Enrichment Analysis (ORA) by using *ClusterProfiler* package to query GO:BP database, with the resulting statistical analyses, to detect altered cellular pathways. In order to select the genes for the enrichment analysis we set the log2FC threshold to 0.5 (| log2FC | ⋝ 0.5) and the adjusted *p* value to 0.05. After filtering the results by *p*-value 0.05 we retrieved 45 statistically deregulated processes among the 15158 tested against GO:BP database.

### Luminex cytokines quantification

Mice were sacrificed after 2 days of treatment (T2) with Vehicle, SR59230A, αPD-L1, and SR59230A + αPD-L1, and tumor mass excised with scissors and homogenized using the Bio-Plex Cell lysis kit (BIO-RAD, California, USA) and the gentleMACS Octo Dissociator (Miltenyi Biotec, Germany). Tumoral lysates were then tested for their cytokines concentrations by Bio-Plex Pro™ assays (BIO-RAD, California, USA) based on Luminex xMAP technology. IFN-γ, TNF-α, IL-2, IL-6, IL-10, GM-CSF, IL-1α, and IL-1β levels (pg/ml) were measured according to the manufacturer’s instructions. Data were acquired on MAGPIX System (Luminex, Texas, USA) and analysis were performed using the Bio-Plex Manager™ software (BIO-RAD, California, USA).

### Flow cytometric analysis

Flow cytometry analysis was performed on tumor cells and immune cell subpopulations of the tumor microenvironment as follows. Briefly, tumors were homogenized with the Tumor dissociation kit mouse (#130-096-730, Miltenyi Biotec) and tumor infiltrating lymphocytes were isolated from the total cells suspension using the CD45 (TIL) MicroBeads mouse (#130-110-618, Miltenyi Biotec) or CD8 (TIL) MicroBeads mouse (#130-116-478, Miltenyi Biotec) through the autoMACS Pro Separator (Miltenyi Biotec) according to the manufacturer’s instructions. Cells were then incubated and stained with various appropriately diluted combinations of different fluorochrome-conjugated antibodies (Supplementary Table 1). Stained cells were analyzed using a MACSQuant Analyzer 10 Flow Cytometer (Miltenyi Biotec®), and data processed using Flowlogic software (Miltenyi Biotec®).

### Immunofluorescence of cells and tissues

Immunofluorescence analysis of murine tumor mass was performed as follows. Samples were rapidly excised, fixed in buffered 4% formaldehyde for 24 h and paraffin-embedded. Histological sections, 5 μm thick, were cut from samples, were deparaffinized and boiled for 10 min in sodium citrate buffer (10 mM, pH 6.0; Bio-Optica) for antigen retrieval and immunostained over night at 4 °C with primary antibodies (anti-PD-L1, ab238697, Abcam; anti-β3-AR, ab94506, Abcam; anti-CD4, 14-9766-82, eBioscience; anti-CD8, 14-0808-82, eBioscience; DBH, ab209487, Abcam). Immune reaction was revealed incubating sections with secondary antibodies Alexa Fluor 488-conjugated IgG or Alexa Fluor 594-conjugated IgG (1:350; Jackson Laboratory). After counterstaining with 4,6-diamidino-2-phenylindole (DAPI), representative images were acquired by an Olympus BX63 microscope coupled to CellSens Dimension Imaging Software version 1.6 (Olympus). The immunofluorescence staining was evaluated through the ImageJ software (NIH, USA) as the fluorescence intensity of protein of interest was normalized to DAPI values.

### Kaplan–Meier and gene correlation analysis in patients with NB

ADRB3 gene correlation with the immune checkpoint genes of CD80, CTLA4, CD276, CD226, CD40LG, 4-1BBL, and correlation between overall (OS) and event-free survival (EFS) probability of patients with NB and levels of ARDB3 mRNA expression were carried out using the gene expression profile of 649 NB specimens generated using 44 K oligonucleotide microarrays. Profiles and clinical data were retrieved from the R2: genomic analysis and visualization platform (http://r2.amc.nl), (Kocak, custom ag44kcwolf, *n* = 649 cases). The last quartile of the distribution was used as expression cut-off value for high versus low ADRB3 gene expression in Kaplan–Meier analysis.

### Statistics

Statistical analysis was performed using GraphPad Prism (GraphPad, San Diego, CA, USA) by one-way or two-way analysis of variance (ANOVA), followed by the Bonferroni post-hoc test or unpaired student t-test. For the in vivo experiments, according to previous studies on the same animal model [12], at least 6 mice per group were needed to guarantee a power of 80%. Values are presented as mean ± SEM. Significance was considered when P was <0.05 and described in each figure legends. Allocation concealment was performed using a randomization procedure (http://www.randomizer.org/).

## Results

### β3-AR antagonism reduces PD-L1 expression in tumor cells of NB-bearing A/J mice

**Fig. 1:**
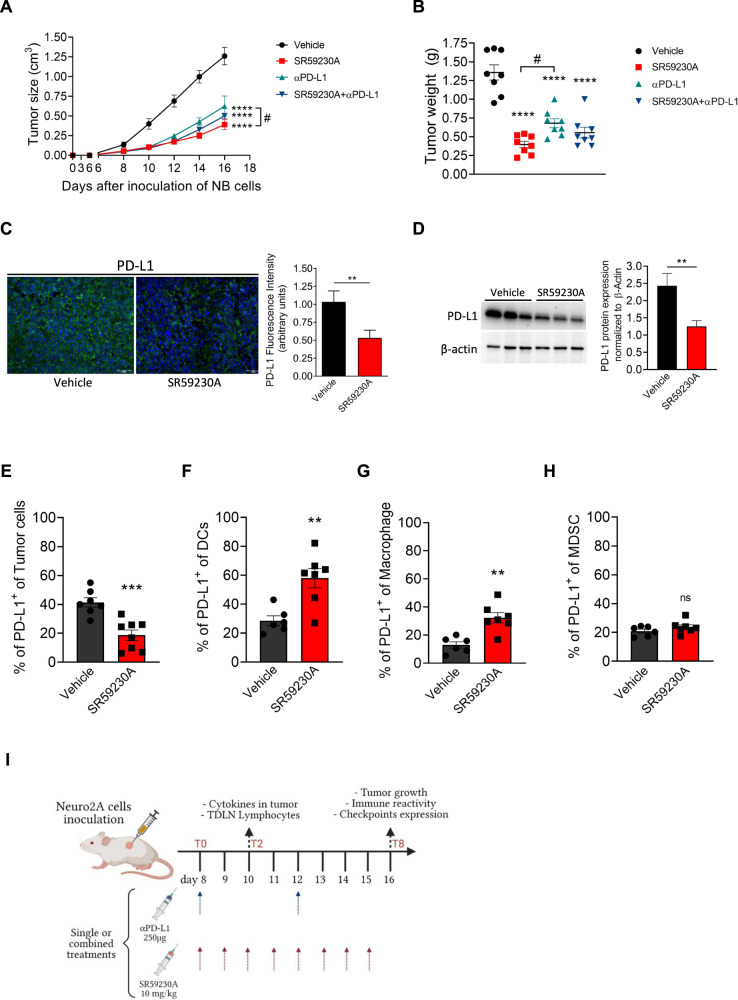
Effect of SR59230A and αPD-L1 treatments on tumor growth and PD-L1 expression in NB tumor-bearing mice. A/J mice were inoculated with N2A tumor cells, and treated from day 8 with SR59230A, αPD-L1 antibody or their combination. **A** Tumor volume and **B** tumor weight were monitored. **C** Immunofluorescence on tumor sections and **D** western blot analysis of total protein lysates of tumor mass performed for evaluation of PD-L1 expression in TME. **E**–**H** Quantification through flow cytometry analysis of PD-L1 expression in tumor, DC, macrophages and MDSC immune cells. **I** Experimental scheme of mice bearing syngeneic N2A cancer cells and treatments with intraperitoneal injections started from day 8 (T0). After 2 days of treatments (T2) mice were sacrificed for evaluation of cytokines in tumor and lymphocytes in tumor draining lymph nodes (TDLNs); after 8 days of treatments (T8) mice were sacrificed for all other analysis (results shown in following figures in paper). Data are expressed as mean ± SEM. **A**, **B** (*n* = 8 per group); **C**, **D** (*n* = 3–4 per group); **E**–**H** (*n* = 7–8 per group). Significance was calculated by one-way (**B**), two-way (**A**) ANOVA analysis followed by Bonferroni’s post hoc test or unpaired student *t*-test (**C**–**H**). **P < 0.01, ***P < 0.001, ***P < 0.0001 vs Vehicle; ^#^*P* < 0.05 SR59230A vs αPD-L1; ns not significant.

Recent studies showed that PD-1/PD-L1 signaling in NB represents a crucial mechanism to limit immune surveillance, and that PD-L1 expression correlates with high levels of tumor markers [15, 16]. Taking into account these and our previous data showing the ability of β3-AR blockade to elicit an immune competent TME in melanoma-bearing mice [13], we wondered whether, in NB tumor, the β3-AR antagonism could lead to immune response activation dependent on PD-L1 signaling involvement. Administration of the β3-AR antagonist, SR59230A, in a murine syngeneic model of NB, reduced tumor growth to a greater extent than an anti-PD-L1 antibody mAb (αPD-L1) treatment, but no synergistic effect was observed when mice were treated with both drugs (Fig. 1A, B). To investigate whether the anti-tumoral effect brought by SR59230A administration relied in part on PD-L1 expression modulation, we assayed PD-L1 expression level in TME of NB-bearing mice. Interestingly, immunofluorescence (Fig. 1C) and western blot analysis (Fig. 1D) on murine tumor mass showed that PD-L1 expression was decreased in TME of mice treated with the β3-AR antagonist compared to vehicle condition. As extensive literature data showed that in TME, in addition to cancer cells, stromal cells such as the antigen-presenting cells (APCs) MDSC, DCs, and macrophages, are the main sources of PD-L1 [17–20], we further investigated where in the TME, the PD-L1 expression level was affected following β3-AR blockade. Cytofluorometric assay revealed that β3-AR antagonism decreased PD-L1 expression in tumor cells (Fig. 1E), while increased its expression level in DCs and macrophages (Fig. 1F, G). No changes were observed in MDSC population (Fig. 1H).

These first results show that β3-AR blockade exerted a significant anti-tumoral activity in a syngeneic model of NB-bearing mice, and that the concomitant administration of PD-L1 antibody did not induce an anti-tumoral synergistic effect. Interestingly, β3-AR pharmacological antagonism modulated PD-L1 expression level in TME, suggesting the involvement of PD-L1 signaling in the anti-tumoral action brought by β3-AR blockade.

### β3-AR antagonism and administration of αPD-L1 stimulate an immune-reactive TME in NB

**Fig. 2:**
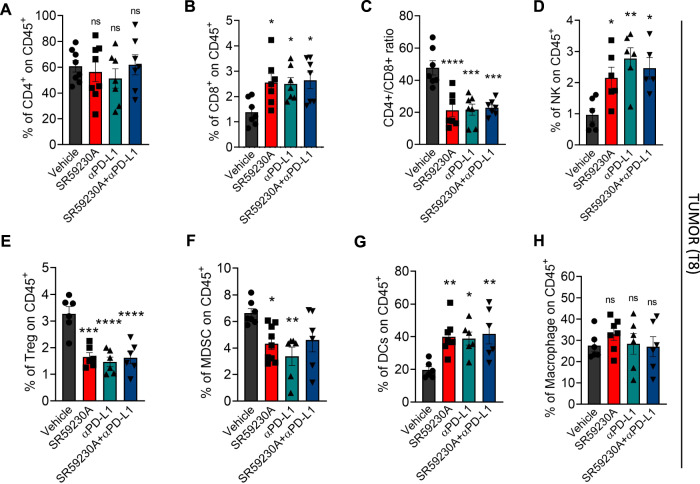
SR59230A and αPD-L1 stimulate an immune-reactive TME in NB. Quantification through flow cytometry analysis of immune cell subpopulations in TME of NB-bearing mice treated for 8 days with SR59230A, αPD-L1 or their combination. **A** CD4^+^ cells (gated on CD45^+^). **B** CD8^+^ cells (gated on CD45^+^). **C** CD4^+^/CD8^+^ ratio. **D** NK cells (NK1.1^+^, NKp46^+^, CD3^−^ gated on CD45^+^). **E** Treg (CD127^-^, CD25^+^, CD4^+^ gated on CD45^+^). **F** MDSC (CD11b^+^, GR1^+^ gated on CD45^+^). **G** DCs (CD11c^+^, F4/80^-^ gated on CD45^+^). **H** Macrophage (F4/80^+^, CD11b^+^ gated on CD45^+^). Data are expressed as mean ± SEM. **A**–**H** (*n* = 8 per group). Significance was calculated by one-way ANOVA analysis followed by Bonferroni’s post hoc test. *P < 0.05, **P < 0.01, ***P < 0.001, ****P < 0.0001 vs Vehicle.

In order to investigate whether the decreased PD-L1 expression, observed in NB tumor cells following β3-AR antagonism, led to an immune reactivation in TME, we first evaluated the number of immune-suppressive and immune-reactive cell subpopulations in tumor mass of A/J NB-bearing mice. TILs were isolated from tumor masses through anti-CD45 microbeads and then stained for cytofluorimetric analysis. Results showed an increased number of anti-tumoral immune cells such as CD8^+^ (with a reduced CD4^+^/CD8^+^ ratio), NK and DCs, and a reduced number of pro-tumoral immune cells such as Treg and MDSC (Fig. 2A–H), in tumor masses of SR59230A-, αPD-L1- and SR59203A + αPD-L1-treated mice compared to vehicle condition.

**Fig. 3:**
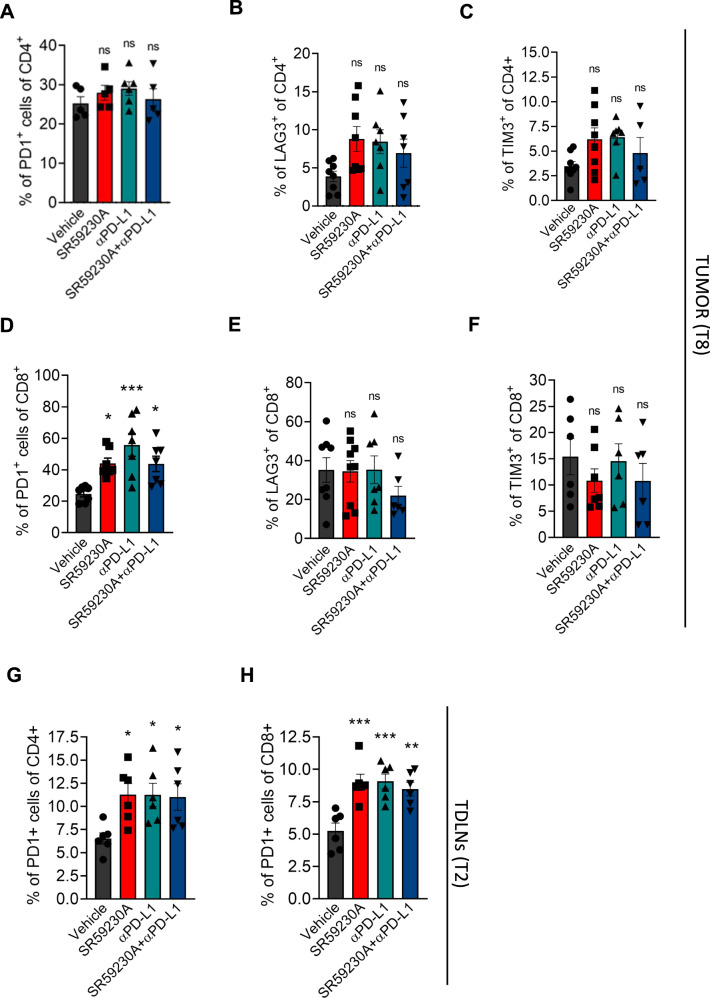
Expression of immune checkpoints on lymphocytes in TME and TDLNs following β3-AR antagonism and administration of αPD-L1. Quantification through flow cytometry analysis of **A** PD1^+^CD4^+^, **B** LAG3^+^CD4^+^, **C** TIM3^+^CD4^+^, **D** PD1^+^CD8^+^, **E** LAG3^+^CD8^+^, **F** TIM3^+^CD8^+^ in tumor mass of NB-bearing mice treated for 8 days with SR59230A, αPD-L1 or their combination, and of **G** PD1^+^CD4^+^ and **H** PD1^+^CD8^+^ in tumor draining lymph nodes (TDLNs) after 2 days of treatment. Data are expressed as mean ± SEM. **A**–**H** (*n* = 5–8 per group). Significance was calculated by one-way ANOVA analysis followed by Bonferroni’s post hoc test. *P < 0.05, **P < 0.01, ***P < 0.001 vs Vehicle; ns not significant.

To characterize the activation of cytotoxic CD4^+^ and CD8^+^ T cells, the main cellular mediators of the anti-tumor immunity, we first assayed the expression of the immune checkpoints PD-1, LAG-3, and TIM-3, known to reflect the immune status of TILs in solid tumors [21]. While both β3-AR antagonism and αPD-L1 treatment, or their combination, did not significantly affect the number of PD1^+^CD4^+^, TIM3^+^CD4^+^, and LAG-3^+^CD4^+^ cells (Fig. 3A–C), neither the number of TIM3^+^CD8^+^ and LAG-3^+^CD8^+^ cells (Fig. 3E, F), they did significantly increase the number of PD1^+^CD8^+^ T cells (Fig. 3D) compared to vehicle in tumor mass of NB-bearing mice after ten days of treatment. Experimental evidences showed that the initial activation/priming of early-effector T cells occurs in tumor-draining lymph nodes (TDLNs) prior to migration of these cells towards the tumor, and that this phenomenon reflects the effectiveness of immune therapies following ICIs administration. Furthermore, upon homing to TDLNs, T cells upregulate PD-1 and proliferate, suggesting PD-1 to be associated with activation rather than T cell exhaustion [22]. We therefore evaluated, during the early phase of tumor formation and after two days of treatment (T2), the number of PD-1^+^CD4^+^ and PD-1^+^CD8^+^ in TDLNs of NB-bearing mice. Results showed a higher number of PD-1^+^ T cells in TDLNs of SR59230A-, αPD-L1- or SR59203A + αPD-L1-treated mice compared to vehicle and no differences among treatments (Fig. 3G, H).

**Fig. 4:**
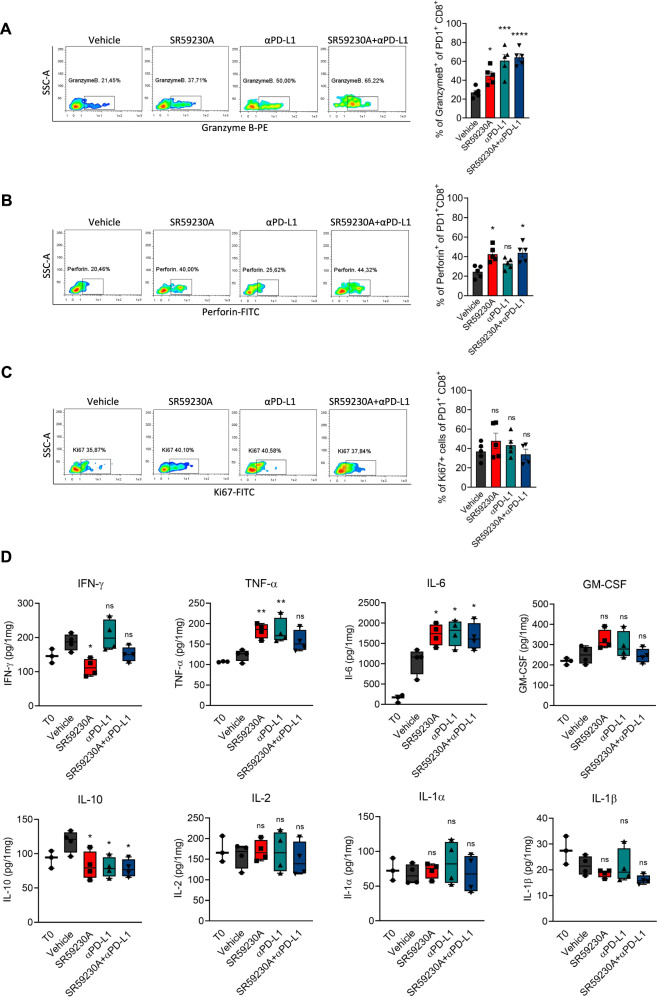
β3-AR blockade triggers CD8 functions and regulates immune cytokines secretion in TME. Representative plots of cytofluorimetric analysis and relative quantification of **A** Granzyme B^+^, **B** Perforin^+^, and **C** Ki67^+^ of PD1 + CD8 + T cells in tumor mass of NB-bearing mice treated for 8 days with SR59230A, αPD-L1 or their combination. **D** Cytokines quantification in tumor mass of NB-bearing mice treated for 2 days with SR59230A, αPD-L1 or their combination. Data are expressed as mean ± SEM. **A**–**C** (*n* = 4–5 per group); **D** (*n* = 4 per group). Significance was calculated by one-way ANOVA analysis followed by Bonferroni’s post hoc test. *P < 0.05, **P < 0.01, ***P < 0.001 vs Vehicle; ^#^*P* < 0.05; ns not significant.

To deeper understand whether the PD-1^+^CD8^+^ cells accumulated in TME of mice treated with the β3-AR antagonist or with the anti-PD-L1 antibody, exerted cytotoxic functions, we measured the production of the effector molecules granzyme B and perforin. Results confirmed an increased production of granzyme B and perforin by PD-1^+^CD8^+^ cells in tumor mass of SR59230A-, αPD-L1- or SR59203A + αPD-L1-treated mice compared to vehicle (Fig. 4A, B). Moreover, a Ki67 staining assay showed a trend indicating an increased proliferation of CD8^+^ T cells in NB tumors of mice treated with the β3-AR antagonist or PD-L1 antibody, even if it failed to reach significant differences (Fig. 4C).

Finally, cytokines measurement showed, in tumor mass of αPD-L1-treated mice, an increased level of cytokines known to exert anti-tumoral actions such as TNF-α, IFN-γ, and IL-6, and a concomitant decrease of the immune-suppressive cytokine IL-10. Surprisingly, in NB-bearing mice treated with the β3-AR antagonist, we observed an increase of TNF-α and IL-6, and decrease of IL-10 levels, according to the immune-reactive status suggested by T cells features, but a decreased level of IFN-γ (Fig. 4D). No significant differences were found in other cytokines assayed (GM-CSF, IL-2, IL-1α, and IL-1β).

Together these data demonstrate that β3-AR antagonism triggers the host immune response in the TME of NB-bearing mice, retracing the same effects observed following PD-L1 antibody administration. In particular, CD8^+^ cytotoxic functions were strengthened by both SR59230A and αPD-L1. No synergistic effects were observed when the β3-AR antagonist was co-administered with the PD-L1 antibody, confirming that the immune reactivation brought by β3-AR blockade relies, at least in part, on the PD-L1 signaling pathway involvement.

### β3-AR on tumor-infiltrating lymphocytes modulates IFN-γ secretion and affects PD-L1 expression on NB tumor cells

**Fig. 5:**
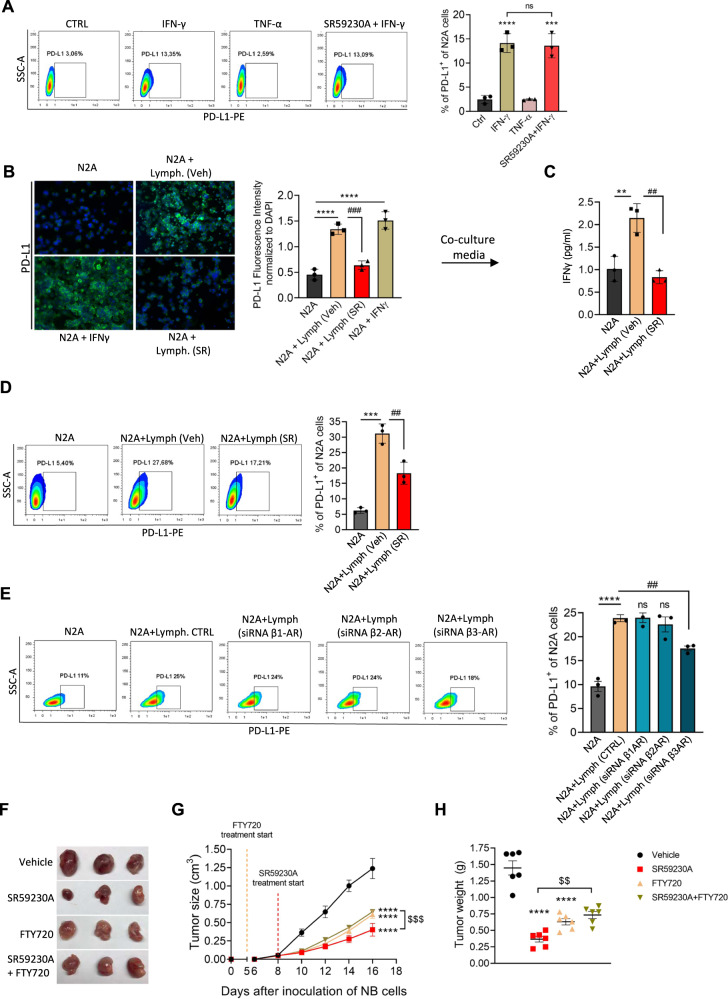
β3-AR on TILs sustains IFN-γ-dependent PD-L1 expression in NB tumor cells. **A** Representative cytofluorimetric plots and relative quantification of PD-L1 expression on N2A tumor cells treated in vitro with IFN-γ (100 ng/mL), TNF-α (100 ng/mL) in presence or not of SR59230A (1 µM) for 48 h. **B** Immunofluorescence and relative quantification of PD-L1 staining on N2A tumor cells co-cultured with lymphocytes obtained from TDLNs of mice treated for 2 days with vehicle or SR59230A. **C** IFN-γ quantification in co-culture medium after 48 h of tumor cells-lymphocytes co-culture. **D** Representative cytofluorimetric plots and relative quantification of PD-L1 expression on N2A tumor cells co-cultured with lymphocytes obtained from TDLNs of mice treated for 2 days with vehicle or SR59230A. **E** Representative cytofluorimetric plots and relative quantification of PD-L1 expression on N2A tumor cells co-cultured with lymphocytes silenced for different β-AR subtypes. **F** Representative images of tumor mass and relative **G** tumor growth and **H** tumor weight of NB obtained from A/J mice inoculated with N2A tumor cells, and treated with SR59230A in presence or not of FTY720. Data are expressed as mean ± SEM. **A**–**E** (*n* = 3 per group); **F**–**H** (*n* = 6 per group). Significance was calculated by one-way (**A**–**E**, **H**) or two-way (**G**) ANOVA analysis followed by Bonferroni’s post hoc test. **P < 0.01, ***P < 0.001, ****P < 0.0001 vs Vehicle; ^##^*P* < 0.01, ^###^*P* < 0.001, ^####^*P* < 0.0001 N2A + Lymph (SR) vs N2A + Lymph (Veh); ^$$^*P* < 0.01, ^$$$^*P* < 0.001 SR59230A vs SR59230A + FTY720; ns not significant.

Expression of PD-L1 on tumor cells can be elicited by several factors, but among others, TNF-α and IFN-γ represent the main cytokines able to increase PD-L1 levels in different tumors [23]. To further elucidate the signaling underlying the β3-AR/PD-L1 cross-talk in our tumor model of NB, we first assayed in vitro the expression of PD-L1 on N2A tumor cells following TNF-α and IFN-γ treatment. Results showed that PD-L1 expression was upregulated in NB cells treated for 48 h with IFN-γ but not with TNF-α. Moreover, pre-treatment of N2A tumor cells with the β3-AR antagonist SR59230A was not able to abrogate the increased PD-L1 expression brought by IFN-γ treatment (Fig. 5A).

Considering that literature data recognized TILs as the main cellular source of IFN-γ during adaptive immune response [24], and that our results revealed that β3-AR modulation on N2A tumor cells did not affect the IFN-γ-dependent PD-L1 expression, we wondered if a β3-AR modulation on TILs was responsible of an altered IFN-γ secretion, which in turn affected the PD-L1 expression on tumor cells. To prove our hypothesis, lymphocytes obtained from TDLNs of NB-bearing mice treated with vehicle or SR59230A were isolated and co-cultured in vitro with tumor cells. Immunofluorescence and flow cytometry analysis confirmed that lymphocytes collected from NB-bearing mice treated with the β3-AR antagonist reduced their ability to trigger the PD-L1 expression on N2A cells compared to untreated lymphocytes (Fig. 5B, D). Furthermore, IFN-γ level measurement in co-culture media confirmed that lymphocytes collected from SR50230A-treated mice, partially lost their ability to secrete IFN-γ when co-cultured with NB tumor cells (Fig. 5C).

It has already been shown that β3-AR may be coupled to Gs protein in several tissues, and its activation leads to activation of cAMP/PKA signaling pathway [25]. At the same time, studies proved the critical role of the cyclic AMP responsive element binding (CREB) as positive transcriptional regulator of IFN-γ in T cells [25–27]. Starting from these observations we assayed whether the cAMP/PKA/CREB signaling pathway was involved in the β3-AR-dependent IFN-γ secretion in TILs. Results showed that β3-AR antagonism of CD8^+^ TILs decreased phosphorylation of PKAα/PKAβ subunits and of CREB, leading to a reduction of IFN-γ expression in CD8^+^ TILs (Supplementary Fig. 1A, B). These results confirmed that β3-AR is able to sustain the IFN-γ transcription in TILs of NB by triggering the cAMP/PKA/CREB signaling pathway. On the other hand, the immunosuppressive cytokine IL-10 was decreased in Treg of mice treated with SR59230A compared to vehicle (Supplementary Fig. 1C), confirming the immune reactivity of the NB TME after β3-AR antagonist treatment of NB-bearing mice.

To confirm the selective effect of the SR59230A antagonism on the β3-AR subtype, we performed a co-culture assay with lymphocytes silenced with siRNAs for β1-, β2- and β3-AR subtypes. Results showed that lymphocytes silenced for β3-AR, among the β-AR subtypes, partially lost their ability to induce PD-L1 expression on N2A tumor cells compared to control condition (Fig. 5E and Supplementary Fig. 1D). Moreover, protein expression of the β3-AR on murine lymphocytes of NB-bearing mice were confirmed by western blot analysis of lymphocytes obtained from TDLNs (Supplementary Fig. 2A) and immunofluorescence of CD4^+^ and CD8^+^ T cells stained for β3-AR on tumor mass sections (Supplementary Fig. 2B).

We previously demonstrated that the specific antagonism of β3-AR on N2A tumor cells by SR59230A, inhibited NB growth and tumor progression, by switching from stemness to differentiation features of tumor cells [12]. Here, our results hinted that the anti-tumoral effect of the β3-AR antagonist administration relied also on the β3-AR signaling modulation on TILs, which in turn led to reinvigoration of immune responses against tumor. Therefore, to corroborate our thesis in vivo, we administered FTY720 to restrain T cells in TDLNs and to reduce their infiltration in tumor mass. FTY720 is an analogue of sphingosine 1-phosphate (S1P), and its administration induces the internalization and degradation of S1P receptor, thus preventing lymphocytes egress from the lymph nodes [28]. As assumed, FTY720 pre-treatment dropped the anti-tumoral effect brought by β3-AR antagonist administration (Fig. 5F–H). To validate the effectiveness of FTY720 to restrain T cells in TDLNs, we measured T cell number in TDLNs after eight days (T8) of treatment. Results showed that both CD4^+^ and CD8^+^ cells were increased in TDLNs of mice pre-treated with FTY720 compared to untreated mice (vehicle) (Supplementary Fig. 2C). According to our and other previous results demonstrating that S1P signaling is crucial for survival of NB tumor cells [12, 29], treatment with FTY720 alone was also able to significantly decrease tumor growth (Fig. 5F–H).

**Fig. 6:**
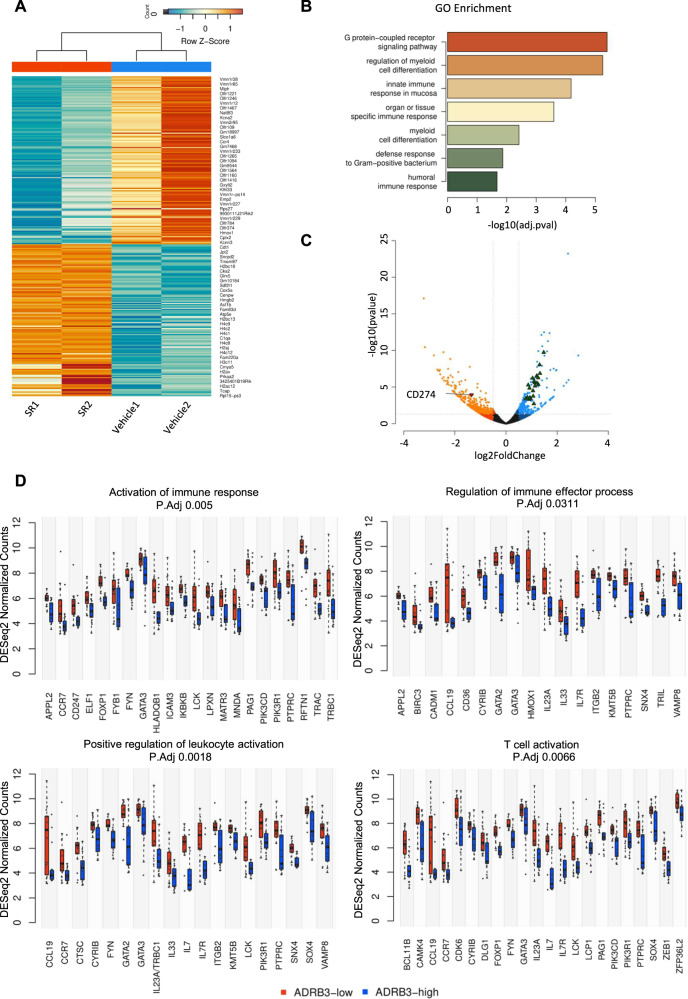
Transcriptomic analyses of NB tumor mass corroborate the prominent role of β3-AR in affecting immune-related processes. RNA sequencing analyses was performed in duplicate in SR59230A- and vehicle-treated NB tumor masses obtained from A/J mice: **A** Heatmap showing the 267 differentially expressed genes (DEGs) which correctly clusterizes replicates as shown in the upper dendrogram; **B** Barplot showing immune-related pathways of ORA results; **C** Volcano plot of gene expression. Lines are set to show the *p* value at 0.05 and the log2FC at 0.5 and represent the thresholds used for ORA. Red and green triangles represent CD274 expression and DEGs in immune-related pathways respectively. **D** Boxplots of DESeq2 normalized counts of genes within the over-represented pathways resulting by comparing ADRB3-high *vs* ADRB3-low expression groups (median value as threshold between groups) with ClusterProfiler in tumor mass of 34 NB patients (data obtained using the public dataset GSE14880 from GEO archive).

RNA sequencing analysis on NB tumor masses isolated from vehicle and SR59230A-treated mice identified 5607 genes among which 267 were differentially expressed genes (DEGs), 127 upregulated and 140 downregulated in SR59230A-treated tumor (Fig. 6A). In particular, several genes that were statistically differentially expressed belonged to immune-related pathways according to GO:BP database (Fig. 6B). Moreover, downregulation of the PD-L1 mRNA (CD274 gene) in SR59230A-treated tumor compared to vehicle condition and upregulation of different other genes involved in immune-related processes (Fig. 6C) corroborated the correlation between β3-AR and the immune-checkpoint PD-L1 observed at protein level. Finally, differential gene expression analysis on a public dataset (GSE14880 from GEO archive) consisting of 34 patients affected by NBs, confirmed that NB tumor masses with a low expression of ADRB3 showed a positive regulation of immune responses, T cell activation, and immune effector pathways compared to tumor mass with high levels of ADRB3 (Fig. 6D).

To note, while in different cancers, catecholamines are released through cancer-associated neurons or simply reach the tumor via blood circulation, in NB excessive levels of catecholamines and their metabolites (HVA, VMA, dopamine), a unique feature of this malignancy, originates from a different source. Indeed, due to the peculiar origin of NB cells (from defective sympathetic neuronal differentiation cells of the neuronal crest), these tumors are usually able to secrete catecholamines by themselves. Indeed, in our NB murine model, immunofluorescence on tumor masses showed that NB tumor cells were positive for the Dopamine β-Hydroxylase (DBH) (Supplementary Fig. 3A), enzyme responsible for the norepinephrine synthesis, regardless of the β3-AR expression on tumor or its antagonism.

Taken together, these data highlight the crucial role played by the β-adrenergic signaling in regulating different biological processes involved in the interaction between the host immune system and the tumor microenvironment in NB tumors. The anti-tumor effects observed following β3-AR antagonist administration in our murine syngeneic model, rely, at least in part, on the modulation of the β3-AR expressed on lymphocytes infiltrating the tumor mass. In particular, we demonstrated that selective blockade of β3-AR on TILs reduced IFN-γ secretion, which in turn decreased the PD-L1 expression on NB tumor cells promoting an immune-reactive TME.

### ADRB3 gene expression correlates with poor prognosis in NB patients

**Fig. 7:**
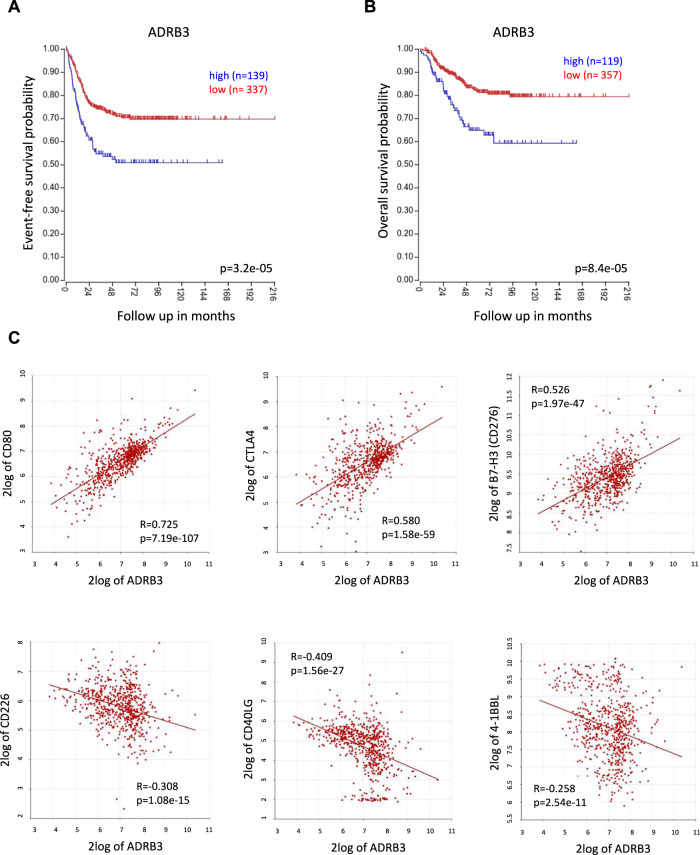
Kaplan–Meier survival analysis and immune checkpoints correlation with *ADRB3* gene expression in NB patients. Kaplan–Meier curves for **A** event-free survival and **B** overall survival of ADRB3 expression (low versus high) in patients affected by NB. **C** Gene correlation analysis between ADRB3 and the immune checkpoints CD80, CTLA4, CD276, CD226, CD40LG and 4-1BBL. Data were obtained using the public dataset R2 (Kocak, custom ag44kcwolf, *n* = 649 cases).

Some studies suggested that β3-AR mRNA and/or protein are overexpressed and in some cases correlated with neoplastic proliferation and transformation in different human cancers [10, 30–32]. Accordingly, our preclinical results obtained in a syngeneic murine model of NB, demonstrated that β3-AR signaling is able to sustain different pro-tumoral processes favoring tumor growth. Therefore, to test whether *ADRB3* gene expression correlates with survival of NB patients, we performed a Kaplan–Meier analysis through the R2: Genomics Analysis and Visualization Platform (http://r2.amc.nl), using the Kocak database which includes complete information about clinical data of 649 NB patients. Results showed that *ADRB3* gene expression was associated with poor outcome in patients with NB. High expression of the *ADRB3* gene was, indeed, significantly associated with reduced event-free (*p* = 3.2e−05) (Fig. 7A) and overall survival (*p* = 8.4e−0.5) (Fig. 7B) of NB patients. Furthermore, a gene correlation analysis in some cohorts, showed the positive correlation between the *ADRB3* and genes for inhibitory immune checkpoints, such as CD80, CTLA4, and CD276, and the negative correlation with genes for immune checkpoints activating the immune system, such as CD266, CD40 ligand (CD40LG) and 4-1BB ligand (4-1BBL) (Fig. 7C).

Overall, data obtained highlight the negative correlation of the *ADRB3* gene expression with the outcome of NB patients, and corroborate and strengthen the role of the β3-AR as molecular player capable of dampening anti-tumor immune responses by affecting multiple immune checkpoint signaling pathways.

## Discussion

Since its peculiar neurogenic origin, high levels of catecholamine metabolites characterize NB tumor condition [7, 33]. In a previous work we speculated that catecholamine release in TME might target cancer cells through the activation of β-ARs, triggering a feedforward loop of growth and malignancy in NB cancer. Indeed, experimental data demonstrated that β3-AR is expressed on different NB tumor cell lines and biopsies from patients, and that its modulation significantly affected tumor growth in a syngeneic murine model of NB [12]. In addition to the β-ARs expression on tumor cells, it is widely known that β-ARs are located on the surface of most cells in TME, including immune cells, and that, catecholamines ligation to β-ARs in immune cells regulate both innate and adaptive immunity [34]. To note, while under physiological conditions, modulation of the immune system by β-adrenergic signaling promotes normal immune response, in altered processes, the SNS/catecholamines/β-ARs-prolonged stimulation may have deleterious effects on different immune cells including lymphocytes [35, 36]. In these altered conditions, a sustained stimulation of the adrenergic signaling promotes pathological processes, and among others, cancer progression [37].

Starting from the aforementioned observations, our study has the intent to investigate whether the anti-tumoral effects brought by β3-AR antagonism in NB tumor, could in part rely on the reinvigoration of the anti-tumor immunity, and whether this could involve the modulation of immune checkpoints signaling pathways.

Preclinical studies suggest that PD-L1 expression in metastatic NB represents a crucial mechanism for limiting immune surveillance [15], and that combined immunotherapy with anti-PD-L1/PD-1 and anti-CD4 antibodies cures syngeneic disseminated NB [38]. Recently, PD-L1 expression was assessed in several pediatric tumors, and above all, PD-L1 staining was associated with inferior survival among patients with NB [16, 39]. Moreover, it was shown that PD-L1-positive tumors were located in the adrenal gland more frequently than PD-L1-negative tumors, and that catecholamine metabolites (NSE, VMA and HVA) tended to be higher in the PD-L1-positive group than in the PD-L1-negative group [16]. These evidences suggested the ability of the PD-1/PD-L1 signaling axis to control tumor immune escape in primary and metastatic NB, however, they did not examine the precise mechanisms underlying the PD-1/PD-L1 signaling regulation.

In order to investigate whether the β3-AR modulation could affect PD-L1 expression in NB, and the consequent immune reactivity in the TME, we treated A/J mice inoculated with the syngeneic N2A NB cells, with the β3-AR antagonist SR59230A, a PD-L1 monoclonal antibody (αPD-L1), or their combination.

First, we observed that β3-AR antagonism was able to reduce NB tumor growth and concomitantly decrease PD-L1 expression in TME of NB-bearing mice. Flow cytometry revealed that the decreased amount of PD-L1 observed in tumor mass of SR59230A-treated mice, mostly reflected the reduced expression on tumor cells. In stark contrary, PD-L1 expression on DCs and macrophages was upregulated. Several scientific evidences assigned to PD-L1 located on both tumor cells and APCs the capacity to dampen T cell activation, partially hindering an effective immune reaction against the tumor [40]. However, recent studies demonstrated that during antigen uptake and cross-presentation of tumor antigens to T cells, a crucial step in the adaptive immunity, DCs and macrophages upregulate PD-L1 thus limiting an excessive activation of T cells and protecting them from cytotoxic activities of T cells [41, 42]. Therefore, we hypothesized that the decreased expression of PD-L1 on tumor cells and its concomitant increase on APCs in SR59230A-treated tumor mass, likely reflected an immune system reinvigoration in TME.

Results confirmed increased numbers of tumor-infiltrating immune-reactive cells (NK, DCs, and CD8^+^), and reduction of tumor-infiltrating immune-suppressive cells (Treg and MDSC). Expression analysis of the immune checkpoint PD-1 on TILs showed a significantly increased number of PD-1^+^CD4^+^ and PD-1^+^CD8^+^ T cells in TDLNs at early stage of tumor development (T2), and of PD-1^+^CD8^+^ in tumor after 8 days (T8) of SR59230A, αPD-L1, or their combination treatment compared to control. An increasing trend was noted also in the number of CD4^+^ T cells stained for TIM3 or LAG3 in tumor of SR59230A, αPD-L1 treatments and their combination, but they failed to reach statistical significance. For CD8^+^ T cells stained for TIM3 or LAG3, we instead observed a big variability.

While some literature data suggested that PD-1, LAG-3, and TIM-3 expressing T cells are exhausted and dysfunctional [43–45], others demonstrated that these immune checkpoints, in particular PD-1, are also upregulated following activation and effector T cell differentiation [46–48], and therefore expression per se does not reflect an exhausted status, rather these markers identify the autologous tumor-reactive repertoire infiltrating different tumors [46–49]. In agreement, deeper investigations in our tumor model confirmed that β3-AR antagonism, as the treatment with the anti-PD-L1 mAb, triggered cytotoxic functions of CD8^+^, as shown by increased number of PD1^+^CD8^+^ cells stained for perforin or granzyme B, corroborating that PD1^+^ TILs found in tumor mass are functionally active.

High infiltration of CD8^+^ cytotoxic T cells in tumor have been shown to correlate with positive prognosis in patients with several malignancies including melanoma, head and neck, breast, colorectal, ovarian, prostatic, and other cancers [50]. In NB, a recent study demonstrated that tumors enriched in tumor infiltrating T cells had a better clinical outcome, independent of other current indicators adopted for tumor staging [51]. Moreover, recent findings demonstrated that the presence of DC and NK cells in tumor mass is positively correlated with both T cell infiltration and the positive clinical outcome of NB patients [52]. All these clinical evidences agree with our preclinical results, in which a TME enriched of NK, DC, and CD8^+^ T cells, observed following the β3-AR antagonism or αPD-L1 administration, was functionally able to control NB tumor growth.

A fundamental aspect of the host immune system challenge against tumor is the release of different cytokines in TME, which are able to sustain or fade anti-tumoral activities. Measurement of cytokines in tumor confirmed, in SR59230A- and αPD-L1-treated mice, a condition shifted towards an immune-reactive TME. However, while among the anti-tumoral cytokines, TNF-α and IL-6 levels resulted both increased, IFN-γ level dropped in tumor mass of β3-AR antagonist-treated mice. Starting from this observation, we hypothesized that modulation of the β3-AR signaling on tumor-infiltrating T cells, in particular on CD4^+^ and CD8^+^, could be responsible of an altered IFN-γ secretion, which in turn affected the PD-L1 expression on tumor cells. Results confirmed our hypothesis. The selective β3-AR antagonism on TILs rendered, indeed, these cells inefficient in inducing the PD-L1 expression on NB tumor cells due to an altered secretion of IFN-γ, and preventing de facto the establishment of an adaptive immune resistance. In TME, IFN-γ orchestrates both pro-tumorigenic and antitumor immune responses, acting as a cytotoxic cytokine together with granzyme B and perforin to hit tumor cells, but also triggering the synthesis of inhibitory immune checkpoints thus stimulating immune-suppressive mechanisms [53]. This bimodal role of IFN-γ in causing opposite effects is dependent on complex mechanisms which are not yet fully understood, however, the amount of IFN-γ secreted seems to be decisive in defining the actions brought by this cytokine [54]. Accordingly, we hypothesize that in our model the decreased amount of IFN-γ brought by β3-AR antagonism on TILs favored immune responses compared to control condition, in which a chronic exposure to IFN-γ forces more immunosuppressive mechanisms.

Among the β-AR subtypes, β2-AR seems to be the most expressed on the surface of a variety of immune cells. However, it should be noted that β3-AR, the last identified member of the β-ARs, has been the least studied up to date. Therefore, to confirm that SR59230A administration brought its effects blocking mainly the β3-AR subtype, we first investigated and confirmed the β3-AR protein expression on lymphocytes of TDLNs and on tumor-infiltrating T cells. We demonstrated the expression of the β3-AR protein in tumor-infiltrating CD4^+^ and CD8^+^, and then through selective β1-, β2-, and β3-ARs silencing, we corroborated the prominent role of the β3-AR subtype, located on lymphocytes, in controlling the IFN-γ-dependent PD-L1 expression on NB tumor cells. To note, extensive data on different tissues and cell models have shown that while the β1- and β2-ARs are both more prone to the phenomenon of homologous desensitization following the prolonged exposure to endogenous catecholamines, the β3-AR subtype was shown not to be, and when, nevertheless, in some cells the β3-ARs desensitization occurred, it was less pronounced as compared to that of β1- and β2-ARs [55].

We then speculate that in NB tumor, a condition characterized by chronic exposure of catecholamines, the β2-AR subtype on TILs, the most expressed physiologically, may undergo downregulation following desensitization, and conversely the β3-AR earns the pivotal role in mediating the catecholamine-induced pro-tumoral effects.

To investigate in humans the pro-tumoral inclination of the β3-AR expression, we finally performed a genetic analysis on 649 NB patients using the open access R2: Genomics Analysis and Visualization Platform. High *ADRB3* gene expression correlated with the worst clinical outcome (overall and event-free survival) of NB patients compared to lower expression; furthermore, the positive *ADRB3* gene correlation with different immune-suppressive checkpoints such as CD80, CTLA4, and B7-H3 (CD276), and the negative correlation with immune-activating checkpoints such as CD266, CD40LG and 4-1BBL confirmed the negative role played by β3-AR in the complex regulation of the immune reactivity in NB. In particular, the positive correlation of the *ADRB3* gene expression with immune-suppressive checkpoints, such as CD276, a NB-associated molecule found in unresponsive NB variants, in which exerts a protective role from an NK cell-mediated lysis [56, 57], or with CTLA4, whose inhibition was shown to be non-redundant in enhancing antitumor T cell killing in NB [58], highlighted the ability of the β3-AR signaling to sustain different mechanisms favoring tumor immune escape. We can speculate that the correlation between high *ADRB3* gene expression and the increased expression of different immune checkpoints could rely on the β3-AR ability to sustain those mechanisms whose overactivation promotes the onset of tumor resistance, such as the chronic exposure to some pro-inflammatory cytokines.

Early studies of checkpoint inhibition in NB have not been always successful. In particular, both in preclinical study models and in humans, the better responses obtained in therapeutic strategies adopting multiple ICIs suggested that targeting different immune checkpoints represents a non-redundant effective anti-tumoral approach in NB [58, 59]. Then, targeting different immunosuppressive pathways could represent the best therapeutic option to avoid tumor resistance of single-targeted therapy. However, the administration of multiple drugs is always problematic, due to heavy side effects and toxicity, and has not always led to desired results. Therefore, a deeper knowledge of the molecular mechanisms involved in the regulation of the complex network of the immune counterpart of the TME, is needed for the development of new immune-oncology approaches. In this context, the here presented results suggest that targeting the β3-AR in NB TME could represent a possible approach to hit multiple non-redundant pro-tumoral signaling pathways. In particular, by affecting both the host immune system, *via* the removal of the barrier induced by immune checkpoints cooperation, and the tumor cell pro-survival pathways, the β3-AR antagonism may represent a promising strategy with, hopefully, therapeutic potential for patients affected by NB malignancies. More studies are still needed to deepen further mechanisms underlying the complex interaction between the host immune system and the tumor counterpart in NB. Nonetheless, in the light of what we have observed, the study of the β-adrenergic signaling in the TME of NB tumors deserves to be taken into consideration.

## Supplementary information

Supplementary Figures and Table Legends

Supplementary Figure 1

Supplementary Figure 2

Supplementary Figure 3

Supplementary Figure 4

Supplementary Table 1

## Data Availability

The datasets generated and analyzed during the current study are available from the corresponding author on reasonable request.
