# Supplementary material for: β3-adrenergic receptor on tumor-infiltrating lymphocytes sustains IFN-γ-dependent PD-L1 expression and impairs anti-tumor immunity in neuroblastoma
Source: Cancer Gene Ther. 2023 Feb 28;30(6):890–904. doi: 10.1038/s41417-023-00599-x (PMC10281870; doi:10.1038/s41417-023-00599-x)
Supplement: Supplementary file 1 — Supplementary Figures and Table Legends [file 41417_2023_599_MOESM1_ESM.docx]

**Supplementary Figure 1. (A)** Western blot analysis of p-PKAα/β, p-CREB, CREB and β-actin proteins expression in CD8^+^ tumor infiltrating lymphocytes isolated from NB-bearing mice following treatment with SR59230A or vehicle for 2 days. **(B)** Representative cytofluorimetric plots and relative quantification of IFNγ in PD1^+^CD8^+^ and **(C)** of IL-10 in Treg isolated from NB-bearing mice following treatment with SR59230A or vehicle for 2 days. Significance was calculated by unpaired student t-test *P < 0.05, **P < 0.01. **(D)** mRNA expression of β-adrenergic subtypes in lymphocytes silenced for β1-, β2- or β3-AR (relative to Figure 5E). Significance was calculated by one-way ANOVA analysis followed by Bonferroni’s post hoc test. **P < 0.01, ***P < 0.001.

**Supplementary Figure 2. (A)** Western blot analysis of β3-AR protein assessed on N2A tumor cells and tumor infiltrating lymphocytes. **(B)** Immunofluorescence showing CD4^+^ and CD8^+^ tumor infiltrating lymphocytes stained for β3-AR. **(C)** Quantification through flow cytometric analysis of CD4^+^ and CD8^+^ T cells restrained in TDLNs of FTY720-treated mice. **A, B** (n= 3 per group); **C** (n= 4 per group). Significance was calculated by one-way ANOVA analysis followed by Bonferroni’s post hoc test. **P < 0.01, ***P < 0.001 vs Vehicle (C); ****P < 0.0001 vs Ctrl, ns= not significant (D).

**Supplementary Figure 3.** Immunofluorescence for the evaluation of Dopamine β-Hydroxylase (DBH) expression on tumor sections from mice injected with N2A wild type (then treated with Vehicle or SR59230A) and with N2A β3-AR knock out.

**Supplementary Figure 4.** Cytofluorimetric gating strategy used for identification of immune cell subpopulations in TME of NB tumor mass.

**Supplementary Table 1.** Antibodies used for flow cytometric analysis**.**
