# Supplementary figures and images for: β3-adrenergic receptor on tumor-infiltrating lymphocytes sustains IFN-γ-dependent PD-L1 expression and impairs anti-tumor immunity in neuroblastoma
Source: Cancer Gene Ther. 2023 Feb 28;30(6):890–904. doi: 10.1038/s41417-023-00599-x (PMC10281870; doi:10.1038/s41417-023-00599-x)

Supplementary Figure 1

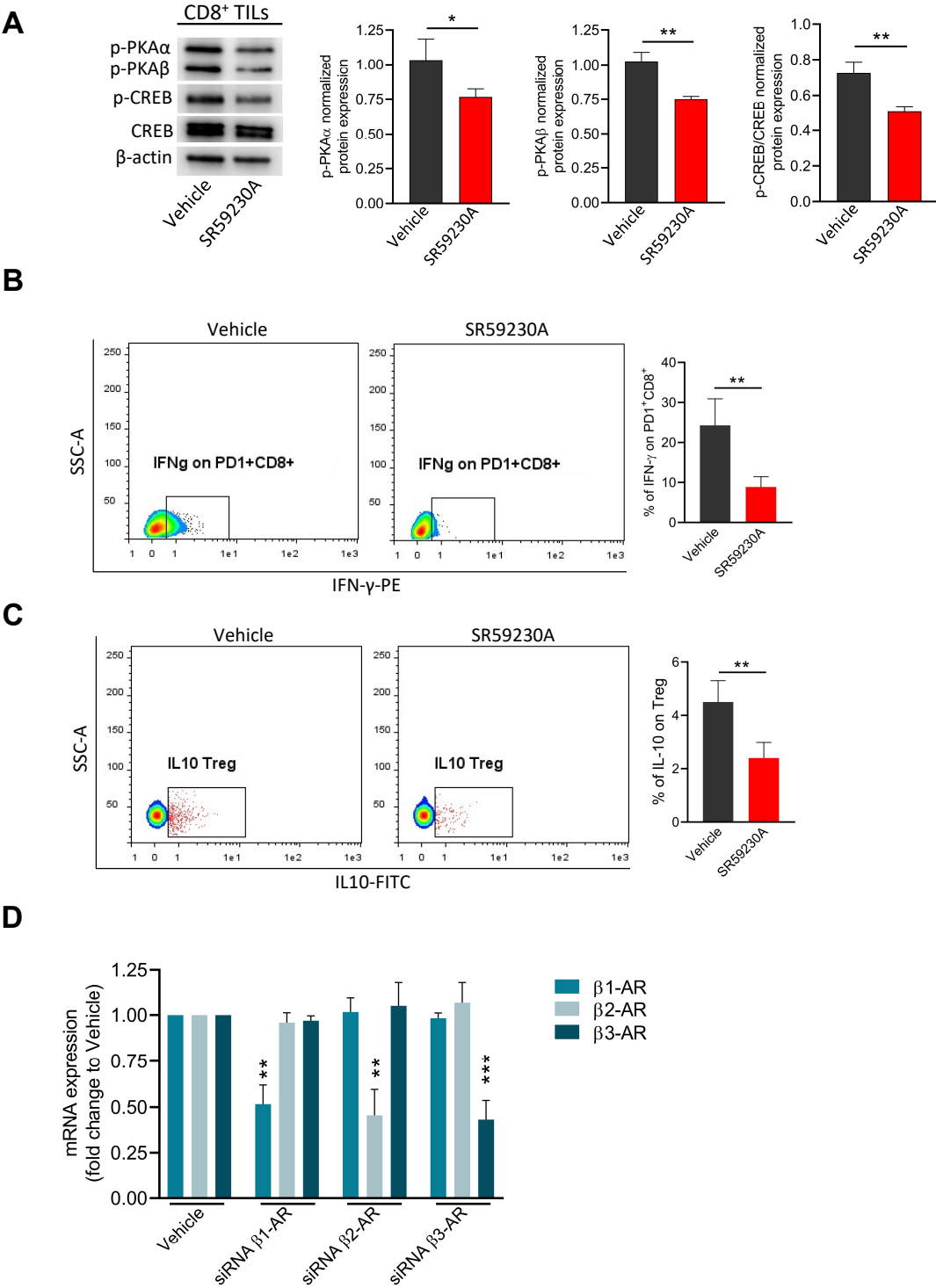

Supplement: Supplementary file 2 — Supplementary Figure 1 [file 41417_2023_599_MOESM2_ESM.pdf]

Supplementary Figure 2

**A**

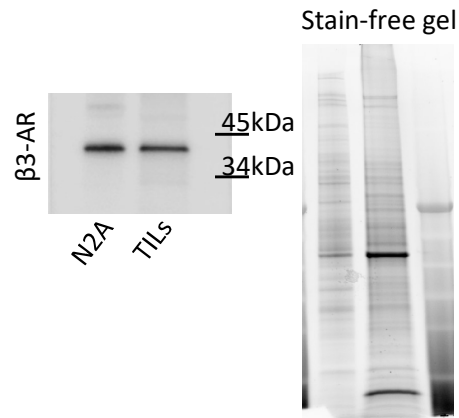

**B**

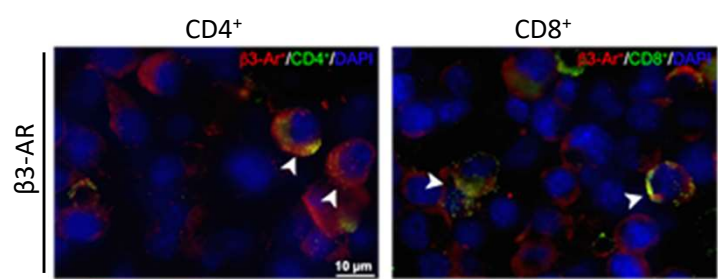

**C**

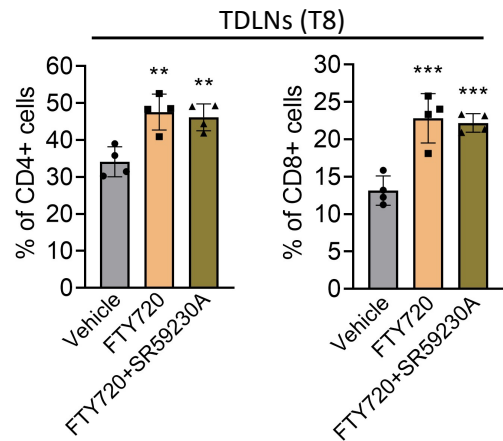

Supplement: Supplementary file 3 — Supplementary Figure 2 [file 41417_2023_599_MOESM3_ESM.pdf]

Supplementary Figure 3

A

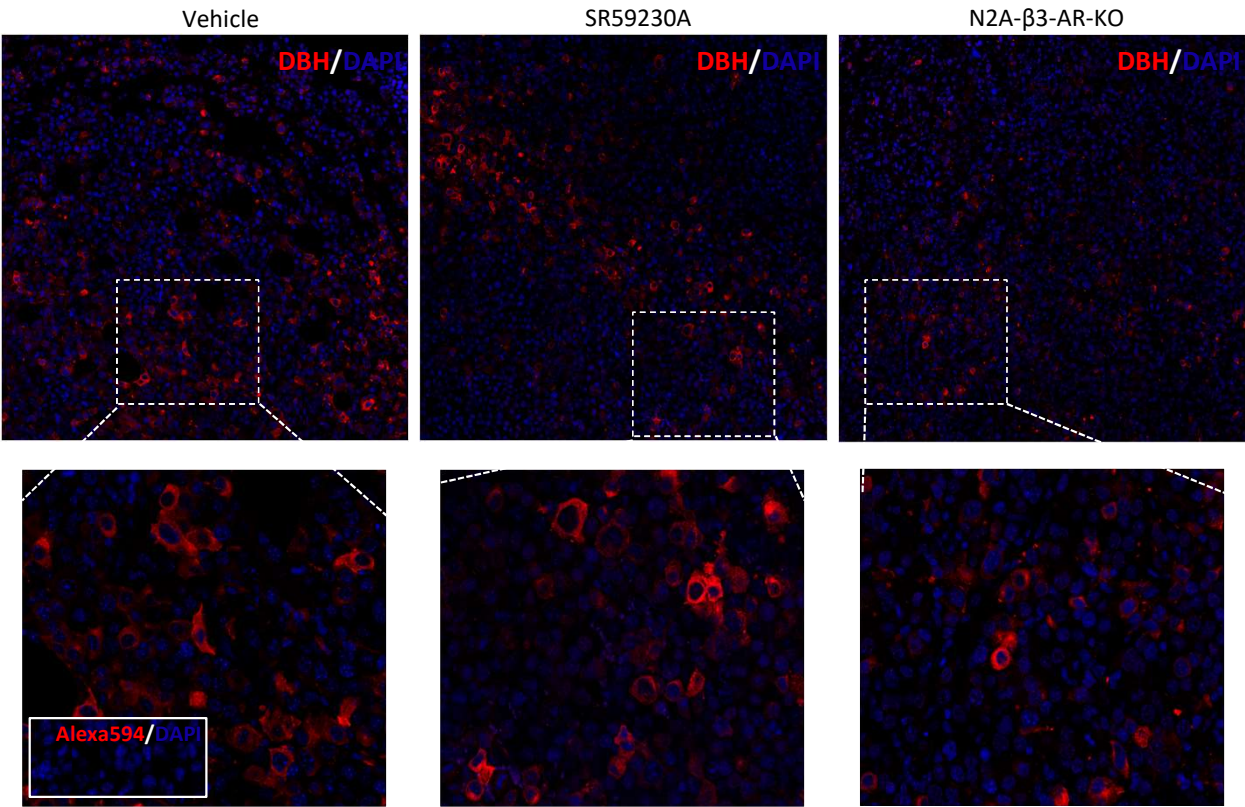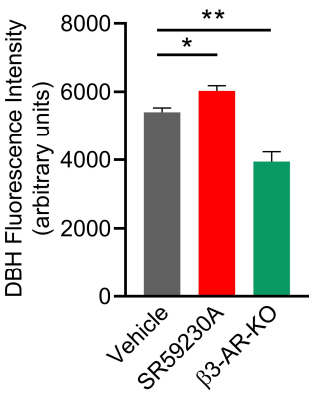

Supplement: Supplementary file 4 — Supplementary Figure 3 [file 41417_2023_599_MOESM4_ESM.pdf]

Supplementary Figure 4

A

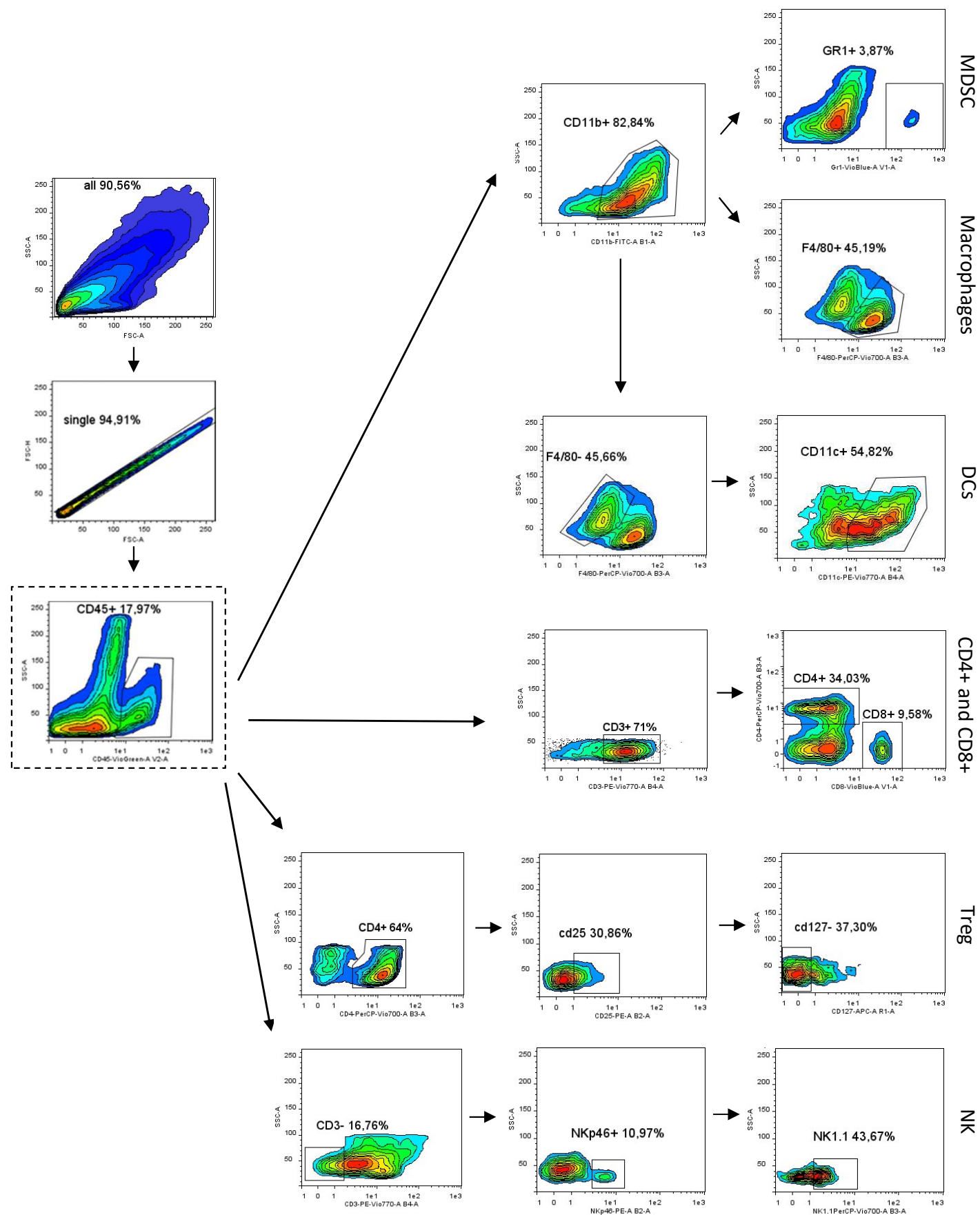

Supplement: Supplementary file 5 — Supplementary Figure 4 [file 41417_2023_599_MOESM5_ESM.pdf]
